# Outcome and prognostic factors of unexpected ovarian carcinomas

**DOI:** 10.1002/cam4.5415

**Published:** 2022-11-10

**Authors:** Ching‐Yu Cheng, Heng‐Cheng Hsu, Yi‐Jou Tai, Ying‐Cheng Chiang, Yu‐Li Chen, Wen‐Fang Cheng

**Affiliations:** ^1^ Department of Obstetrics and Gynecology, College of Medicine National Taiwan University Taipei Taiwan; ^2^ Department of Obstetrics and Gynecology National Taiwan University Hospital Hsin‐Chu Branch Hsinchu City Taiwan; ^3^ Department of Obstetrics and Gynecology Fu Jen Catholic University Hospital New Taipei City Taiwan; ^4^ Department of Surgery National Taiwan University Cancer Center Taipei Taiwan; ^5^ Graduate Institute of Clinical Medicine, College of Medicine National Taiwan University Taipei Taiwan; ^6^ Graduate Institute of Oncology, College of Medicine National Taiwan University Taipei Taiwan

**Keywords:** chemotherapy, laparoscope, laparotomy, outcome, ovarian cancer, prognostic factor, unexpected ovarian cancer

## Abstract

**Background:**

We investigated risk factors influencing the outcome of unexpected ovarian carcinomas.

**Methods:**

We reviewed the ovarian carcinoma patients treated at atertiary medical institution between 2000 and 2017 and analyze the clinico‐pathological characteristics, treatment strategies, recurrence status, and outcome.

**Results:**

A total of 112 women (65 primary laparoscopic surgery [LSC] and 47 laparotomic surgery [LAPA]) were included in the analysis. The LSC group had smaller ovarian tumors (10.5 ± 7.3 cm vs. 16.6 ± 8.7 cm, *p* = 0.031) and higher incidence of subsequent staging surgery (56.9% vs. 25.5%, *p* = 0.0001) compared to the LAPA group. There were 98/112 (86.6%) of early stages (I/II) diseases. The difference between the recurrent rate (27.7% vs. 31.9%), disease‐free survival (DFS), and overall survival (OS) were not significant among surgical groups. In the multivariate analysis, FIGO stage (stage II hazard ratio [HR] 6.61, *p* = 0.007; stage III HR 8.40, *p* = 0.002) was the only prognostic factor for DFS. FIGO stage (stage II HR 20.78, *p* = 0.0001; stage III HR 7.99, *p* = 0.017), histological type (mucinous HR 12.49, *p* = 0.036), and tumor grade (grade 3 HR 35.01, *p* = 0.003) were independent prognostic factors for OS, while women with latency >28 days from primary to staging surgery had significantly poorer OS (*p* = 0.008). Women with latency >28 days between primary surgery and adjuvant chemotherapy had similar DFS (*p* = 0.31) and a trend of poorer OS (*p* = 0.064).

**Conclusions:**

The prognosis of unexpected ovarian cancer is independent from the primary surgical procedure and comprehensive staging surgery should be performed at close proximity after the diagnosis of unexpected ovarian malignancy.

## Abstract 2

Unexpected malignant ovarian cancers were occasionally diagnosed after surgery for the initial diagnosis of benign ovarian tumors. The primary surgical procedure is not a prognostic factor for incidental ovarian cancer. Comprehensive staging surgery should be performed as soon as possible after the diagnosis of incidental ovarian malignancy.

## 1 INTRODUCTION

Epithelial ovarian carcinoma (EOC) is the seventh most common cancer in women and has a worldwide 5‐year survival rate lower than 45%.^1^, ^2^, ^3^ Ovarian cancer is now the eighth leading cause of cancer‐related deaths in women in Taiwan.^4^, ^5^ The incidence in Taiwan is comparable to that of Western countries.^5^ However, in Taiwan, the incidence of EOC has increased and the age of diagnosis decreased in the past two decades. Although the incidence of EOC in Taiwan is comparable to other Western countries, it is on the rise with a younger age of diagnosis in the past 2 decades.^5^ Thus, EOC is an important cancer that needs to be emphasized globally.

Identifying the nature of an ovarian tumor includes physical and pelvic examination, sonography, and imaging studies, such as computerized tomography and magnetic resonance imaging. Laparoscopic surgery (LSC) is the first choice when ovary measures <8 cm. LSC adnexectomy can be used if there are no signs of extra‐ovarian dissemination, which would require immediate mid‐line laparotomy.^6^ Nevertheless, some unexpected ovarian cancers remain undiagnosed during, or even after surgery. The diagnosis of benign or malignant ovarian tumors is achieved by intraoperative frozen and final paraffin pathology.

LSC is the first‐choice surgical procedure for the management of ovarian tumors. LSC became the standard surgical procedure for benign adnexal masses due to its benefits of minimal invasiveness, decreased morbidity, including shorter hospitalization and quicker recovery, and its similar outcomes as conventional laparotomy.^7^ Matsushita et al. reported that, even after strict selection of women with adnexal tumors, unexpected ovarian cancers were noted during or after primary surgery for adnexal tumors.^8^ Due to difficulties in performing differential diagnosis between benign ovarian tumors and early‐stage ovarian cancers before surgery and concerns of potential risks for undertreating ovarian cancer owing to increasing use of minimal access surgery,^9^, ^10^ rate of diagnosing unexpected malignant ovarian cancers varied from 11% to 19% after surgery for the initial diagnosis of benign ovarian tumors, depending on the surgeons' knowledge and the choice of surgical procedures.^11^, ^12^, ^13^, ^14^

Intraoperative rupture of adnexal or ovarian tumors was common during surgery, especially in LSC, and the consequences of the rupture of unexpected ovarian malignancy include upstaging and the need of further surgery and potentially adjuvant therapy, such as chemotherapy.^15^ This potentially worsens the patient's outcome. We conducted a retrospective study to evaluate and identify whether clinico‐pathological characteristics of unexpected ovarian cancers, and the primary surgical procedure method, with or without complete staging surgery, and the interval between the primary surgical procedure and staging surgery or adjuvant chemotherapy influence the outcomes of unexpected ovarian cancer patients.

## 2 MATERIALS AND METHODS

### 2.1 Study population

The medical records of patients diagnosed with EOC at National Taiwan University Hospital (NTUH) between January 1, 2000, and December 31, 2017, were retrospectively reviewed. Fertility‐sparing surgery to preserve the uterus and/or unilateral ovary was performed for some patients who did not complete their family planning. The study protocol was approved by the Institutional Review Boards of the hospital. These EOC patients were eligible for inclusion in the study if they met the following criteria: adnexal LSC/LAPA with unilateral or bilateral ovarian cystectomy, oophorectomy, or salpingo‐oophorectomy with or without hysterectomy; no intraoperative frozen pathology; underwent follow‐up, adjuvant chemotherapy, and/or staging or debulking surgery, including pelvic and/or para‐aortic lymph node sampling or dissection, and infracolic‐omentectomy; histology was reviewed by proficient pathologists specialized in gynecologic oncology; and available prognosis data, such as clinico‐pathological and survival data. The exclusion criteria included final benign or borderline pathologies or frozen pathology during surgery, and lost follow‐up after surgery.

### 2.2 Data collection

The patient demographic and clinical data, including age at diagnosis, tumor histology and grade, FIGO stage, type of chemotherapy, and follow‐up data were extracted from medical records and stored in a database. The International Federation of Gynecology and Obstetrics (FIGO) 2014 classification was used for disease staging and histological grading.^16^ If the patient did not undergo staging surgery, the FIGO stage would be based on post‐surgical imaging. The regimens for front‐line adjuvant chemotherapy were: PT (carboplatin + paclitaxel/cisplatin + paclitaxel/carboplatin + docetaxel) and CP (cyclophosphamide + carboplatin/cyclophosphamide + cisplatin). The choice of chemotherapeutic regimen with platinum and cyclophosphamide or paclitaxel was made by the patients' informed decision with the physician and their financial condition as paclitaxel is not reimbursed by the national health insurance in Taiwan for patients in FIGO stage I/II. All patients with advanced stages (stage IIb/III) received 6–9 cycles of platinum‐based adjuvant chemotherapy combined with paclitaxel regimens. For early‐stage (stages I to IIa) patients, the Taiwan Gynecologic Oncologic Group (TGOG) of the National Health Research Institute (NHRI) of Taiwan guidelines recommended 3–6 cycles of chemotherapeutic regimen, based on the physicians' input and the patients' tolerance to the side effects. The recurrence of EOC was confirmed by radiology, physical findings, tissue biopsy, and elevation of twofold of the upper normal limit in serum carcinoma antigen 125 (CA‐125) or carcinoembryonic antigen (CEA) in two consecutive tests within a 2‐week interval.

### 2.3 Statistical analysis

Overall survival (OS) was defined as the time from the date of primary surgery to the death from any cause or censored at the last recorded clinical visit. Disease‐free survival (DFS) was defined as the time interval from the last date of treatment (chemotherapy or surgery) to clinically defined recurrence, death from any cause, or the last recorded clinical visit, whichever occurred first. The Kaplan–Meier method was used to estimate the DFS and OS between treatment groups, and the differences in survival curves between the regimens were tested by the log‐rank test. We used univariate and multivariate Cox proportional hazard regression models to examine the effects of each pathological feature and surgical method on recurrence and survival, and to calculate the adjusted hazard ratios (HRs) and 95% confidence intervals (CIs). Trend of an independent variable was evaluated by the standard log‐rank test. All statistical analyses were performed in SPSS 23 (SPSS, Inc.) and all statistical tests were two‐sided with *p* < 0.05, indicating significance.

## 3 RESULTS

### 3.1 Patients

We identified 112 women with unexpected EOC based on the inclusion and exclusion criteria. The incidence of unexpected ovarian cancer was 4.7% in our department (from 2000 to 2017, there were total of 2383 ovarian cancer cases in our institute). The clinico‐pathological characteristics are provided in Table 1. Average follow‐up period for all patients was78.5 months. Average age at diagnosis was 42.4 years, with 75.9%of the women under the age of 50. Ninety‐eight women (86.6%) had early‐stage (stage I/II) disease. Clear cell histology accounted for 32.1% of the patients, followed by mucinous carcinoma (30.4%), endometrioid (16.1%), and serous (15.2%). Seventy‐five women (66.9%) received adjuvant chemotherapy after diagnosis and 49 (43.8%) women underwent staging or debulking surgery after the primary surgery. More women received subsequent staging surgery post‐LSC compared to post‐LAPA (61.5% vs. 32.5%, *p* = 0.001, chi‐squared test). Thirty‐three women (27.7%) who underwent LSC and 25 (22.3%) who underwent LAPA experienced recurrence and disease‐related death during follow‐up.

**TABLE 1:** Basic characteristics of 112 incidental EOC women

Characteristics	Total (*N* = 112)		LSC (*N* = 65)		LAPA (*N* = 47)		*p*
	Number	%	Number	%	Number	%	
Age(mean ± SD)	42.4 (13.5)		43.0 (13.1)		41.6(14.1)		0.55
Parity[mean ± SD]	0.9 (1.2)		0.9 (1.2)		0.8 (1.2)		0.56
Tumor Size (cm) [mean ± SD]	13.2 (8.5)		10.5 (7.3)		16.6 (8.7)		**0.031**
FIGO stage^c^							0.051
Ia + Ib	35	31.3%	15	23.1%	20	42.6%	
Ic	55	49.1%	37	56.9%	18	38.3%	
II	8	7.1%	4	6.2%	4	8.5%	
III	14	12.5%	9	13.8%	5	10.6%	
Histology							0.43
Serous	17	15.2%	12	18.5%	5	10.6%	
Endometrioid	18	16.1%	11	16.9%	7	14.9%	
Mucinous	34	30.4%	16	24.6%	18	38.3%	
Clear cell	36	32.1%	20	30.8%	16	34.0%	
Others^a^	7	6.2%	6	9.2%	1	2.2%	
Grade							0.58
I	27	24.1%	16	24.6%	11	23.4%	
II	2	1.8%	2	3.1%	0	0.0%	
III	54	48.2%	32	49.2%	22	46.8%	
N/A	29	25.9%	15	23.1%	14	29.8%	
Adjuvant C/T							0.31
Yes	75	66.9%	46	70.8%	29	61.7%	
No	37	33.1%	19	29.2%	18	38.3%	
Staging surgery							**0.001**
Yes	49	43.8%	37	56.9%	12	25.5%	
No	63	56.2%	28	43.1%	34	74.5%	
Recurrence							0.26
Yes^b^	33	29.5%	18	27.7%	15	31.9%	
No	79	70.5%	47	72.3%	32	68.1%	
Death							0.25
Yes	25	22.3%	12	18.5%	13	27.7%	
No	87	77.7%	53	81.5%	34	72.3%	

Abbreviations: C/T, chemotherapy; EOC, epithelial ovarian carcinoma; LAPA, laparotomy; LSC, laparoscope; N, number; N/A, not available; SD, standard deviation.

^a^ 1 case of neuroendocrine cell, 1 case of transitional cell carcinoma, 3 cases of adenocarcinoma, 1 case of squamous cell carcinoma, 1 case of MMMT.

^b^ Including 4 persistent diseases in LSC group and one in LAPA group.

^c^ Ia: 24 incomplete staging, Ib; 1 incomplete staging, Ic; 30 incomplete staging, II: 4 incomplete staging, III: 4 incomplete staging.

### 3.2 Prognostic factors for DFS

We analyzed the prognostic factors influencing DFS (Table 2). FIGO stage (stage Ic HR 1.77, 95% CI 0.60–5.18, *p* = 0.30; stage II HR 6.61, 95% CI 1.69–25.84, *p* = 0.007; stage III HR 8.40, 95% CI 2.24–31.50, *p* = 0.002) was the only prognostic factor for DFS in multivariate analysis (when compared to FIGO stage Ia + Ib). Other factors, such as age, histology, tumor grade, primary surgical procedure, staging surgery, and adjuvant chemotherapy were not confounding factors in incidental ovarian carcinoma.

**TABLE 2:** Prognostic factors of disease‐free survival of 112 incidental EOC women by Cox regression analysis

	Univariate			Multivariate		
	HR	95% CI	*p*	HR	95% CI	*p*
Age						
<50	1.00	(Reference)		1.00	(Reference)	
≥50	1.1	0.51–2.37	0.81	0.73	0.32–1.88	0.57
FIGO stage						
Ia + Ib	1.00	(Reference)		1.00	(Reference)	
Ic	1.87	0.67–5.18	0.23	1.77	0.60–5.18	0.30
II	5.56	1.61–19.25	**0.007**	6.61	1.69–25.84	**0.007**
III	7.04	2.35–21.07	**<0.001**	8.40	2.24–31.50	**0.002**
Histology						
Serous	1.00	(Reference)		1.00	(Reference)	
Endometrioid	0.65	0.22–1.87	0.42	0.89	0.18–4.48	0.89
Mucinous	0.48	0.18–1.24	0.13	0.96	0.19–4.93	0.96
Clear cell	0.47	0.18–1.21	0.12	0.88	0.27–2.91	0.83
Others^a^	0.38	0.05–3.04	0.36	0.71	0.065–7.87	0.78
Grade						
I & II	1.00	(Reference)		1.00	(Reference)	
III	2.23	0.82–6.07	0.12	2.63	0.44–15.77	0.29
Primary surgery method						
LSC	1.00	(Reference)		1.00	(Reference)	
LAPA	1.04	0.52–2.07	0.92	1.04	0.48–2.26	0.91
Staging surgery						
No	1.00	(Reference)		1.00	(Reference)	
Yes	1.09	0.75–1.59	0.64	0.82	0.35–1.93	0.64
Adjuvant C/T						
No	1.00	(Reference)		1.00	(Reference)	
Yes	1.39	0.65–2.99	0.40	1.04	0.42–2.55	0.94

Abbreviations: C/T, chemotherapy; CI, confidence interval; EOC, epithelial ovarian carcinoma; LSC, laparoscope; LAPA, laparotomy.

^a^ Including 1 case of neuroendocrine carcinoma, 1 case of transitional cell carcinoma, 3 cases of adenocarcinomas, 1 case of squamous cell carcinoma, 1 case of mixed malignant Mullerian tumor.

### 3.3 Prognostic factors for OS

We also analyzed the prognostic factors influencing OS (Table 3). Independent prognostic factors: FIGO stage (stage II HR 20.78, 95% CI 4.09–105.49, *p* = 0.0001; stage III HR 7.99, 95% CI 1.45–44.05, *p* = 0.017), histological type (mucinous HR 12.49, 95% CI 1.18–132.12, *p* = 0.036), and tumor grade (grade 3 HR 35.01, 95% CI 3.42–30.59, *p* = 0.003) were associated with poor OS in multivariate analysis (compared to stage Ia + Ib, grade I/II, or serous histology). Age, primary surgical procedure, staging surgery, and adjuvant chemotherapy were not prognostic factors for OS in unexpected ovarian carcinoma.

**TABLE 3:** Prognostic factors for overall survival of 112 incidental EOC women by Cox regression analysis

	Univariate			Multivariate		
	HR	95% CI	*p*	HR	95% CI	*p*
Age						
<50	1.00	(Reference)		1.00	(Reference)	
≥50	0.89	0.35–2.22	0.79	0.55	0.18–1.68	0.29
FIGO stage						
Ia + Ib	1.00	(Reference)		1.00	(Reference)	
Ic	2.11	0.58–7.67	0.26	2.39	0.60–9.54	0.22
II	8.72	2.08–36.57	**0.003**	20.78	4.09–105.49	**<0.001**
III	8.49	2.17–33.21	**0.002**	7.99	1.45–44.05	**0.017**
Histology						
Serous	1.00	(Reference)		1.00	(Reference)	
Endometrioid	0.44	0.11–1.76	0.25	2.29	0.42–12.345	0.34
Mucinous	0.61	0.21–1.76	0.36	12.49	1.18–132.12	**0.036**
Clear cell	0.45	0.15–1.4	0.17	0.79	0.18–3.42	0.75
Others^a^	1.22	0.25–6.07	0.81	15.64	1.24–196.98	**0.033**
Grade						
I & II	1.00	(Reference)		1.00	(Reference)	
III	4.64	1.05–20.42	**0.043**	35.01	3.42–30.59	**0.003**
Primary surgery method						
LSC	1.00	(Reference)		1.00	(Reference)	
LAPA	1.21	0.55–2.65	0.64	1.17	0.48–2.89	0.73
Staging surgery						
No	1.00	(Reference)		1.00	(Reference)	
Yes	1.65	0.75–3.63	0.22	1.79	0.66–4.89	0.25
Adjuvant C/T						
No	1.00	(Reference)		1.00	(Reference)	
Yes	1.25	0.52–2.99	0.62	0.69	0.22–2.19	0.54

Abbreviations: C/T: chemotherapy; CI: confidence interval; EOC, epithelial ovarian carcinoma; LSC: laparoscope; LAPA: laparotomy.

^a^ Including 1 case of neuroendocrine cell, 1 case of transitional cell carcinoma, 3 cases of adenocarcinoma, 1 case of squamous cell carcinoma, 1 case of MMMT.

### 3.4 Treatment strategy and outcome

**FIGURE 1:**
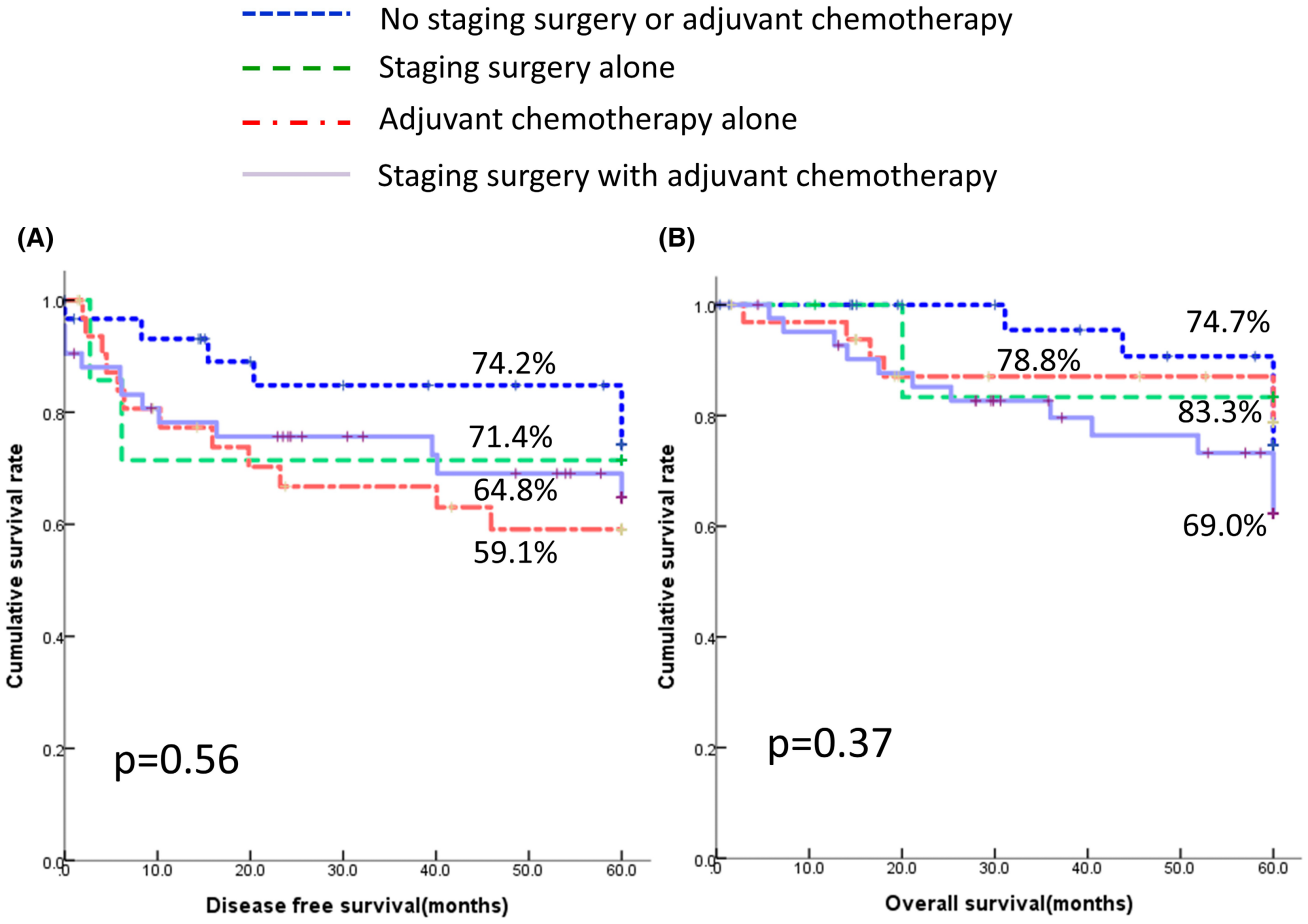
Survival curves for 112 women with unexpected EOC receiving different treatment modalities. (A) 5‐year disease‐free survival (DFS). No staging surgery or adjuvant chemotherapy 74.2% (95% CI 64.9–83.5), staging surgery alone 71.4% (95% CI 54.3–88.5), adjuvant chemotherapy alone 59.1% (95% CI 49.9–68.3), staging surgery with adjuvant chemotherapy 64.8% (95% CI 56.6–73.0), *p* = 0.56. (B) 5‐yearoverall survival (OS). No staging surgery or adjuvant chemotherapy 74.7% (95% CI 64.8–84.6), staging surgery alone 83.3% (95% CI 68.1–98.5), adjuvant chemotherapy alone 78.8% (95% CI 71.0–86.6), staging surgery with adjuvant chemotherapy 62.2% (95% CI 53.6–70.8), *p* = 0.37.

Next, we analyzed whether different treatment modalities influence the outcome of unexpected ovarian cancer. As demonstrated in Figure 1A, there were no differences in DFS (*p* = 0.56, log‐rank test) and OS (*p* = 0.37, log‐rank test; Figure 1B) among the four groups: women who received observation alone (no staging surgery or adjuvant chemotherapy), staging surgery alone, adjuvant chemotherapy alone, or staging surgery with adjuvant chemotherapy.

### 3.5 Surgical latency

**FIGURE 2:**
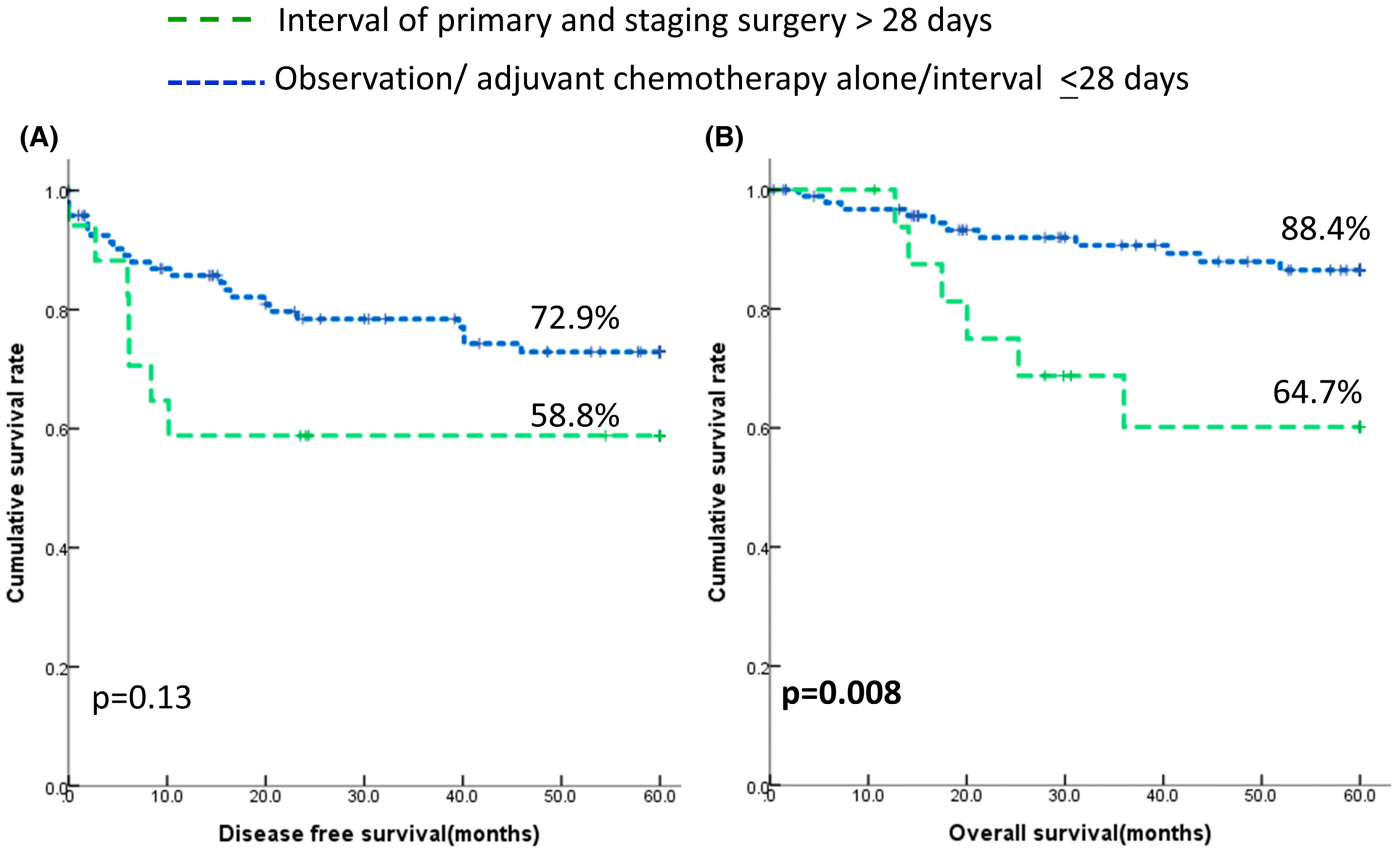
Influence of surgical latency between primary and staging surgeries on the outcome of unexpected EOC in 112 women. (A) 5‐year disease‐free survival (DFS). Observation, staging <28 days, or chemotherapy alone72.9% (95% CI 68.0–77.8). Staging >28 days 58.8% (95% CI 46.9–70.7), *p* = 0.13. (B) 5‐year overall survival (OS). Observation, staging <28 days, or chemotherapy alone 86.5% (95% CI 82.7–90.3). Staging >28 days 60.2% (95% CI 47.3–73.1), *p* = 0.008.

We further evaluated whether latency between the primary surgery and staging surgery influences the outcome of unexpected ovarian carcinoma. Shown in Figure 2A, women who had surgical latency >28 days between the primary and staging surgery had a trend of shorter DFS than observation alone, chemotherapy alone, or surgical latency ≤28 days, but were not significant (*p* = 0.13, log‐rank test). However, those with surgical latency >28 days had significantly poorer OS than those who underwent observation alone, chemotherapy alone, or surgical latency ≤28 days (*p* = 0.008, log‐rank test).

Our results indicated that surgical latency influences the outcome of unexpected ovarian cancer.

### 3.6 Chemotherapy latency

**FIGURE 3:**
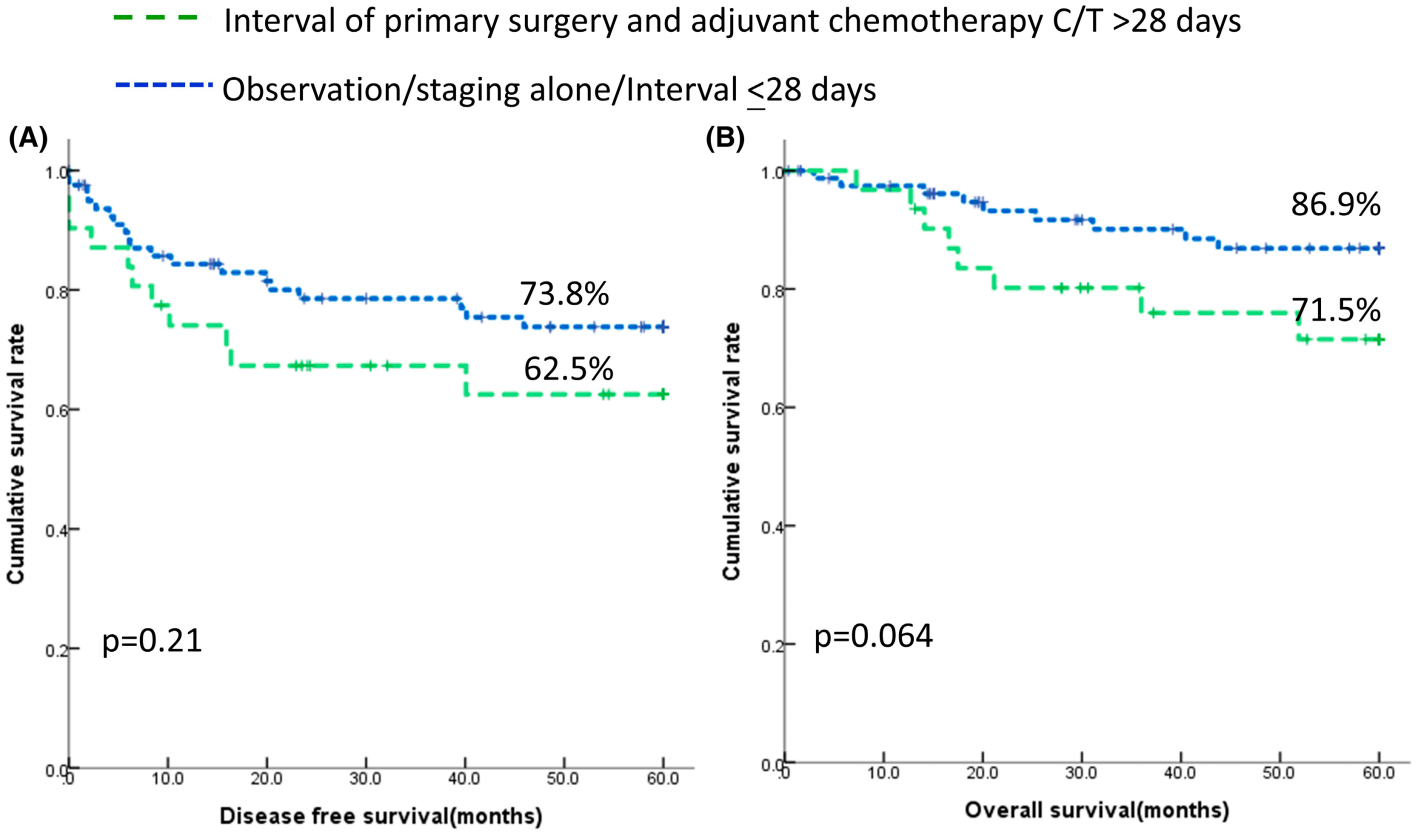
Influence of chemotherapy latency between primary surgery and adjuvant chemotherapy on the outcome of unexpected EOC in 112 women. (A) 5‐year disease‐free survival (DFS). Observation, chemotherapy <28 days, or staging alone 73.8% (95% CI 68.6–79.0). Chemotherapy >28 days 62.5% (95% CI 53.3–71.7), *p* = 0.21. (B) 5‐yearoverall survival (OS). Observation, chemotherapy <28 days, or staging alone 86.9% (95% CI 82.8–91.0). Chemotherapy >28 days 71.5% (95% CI 62.8–80.2), *p* = 0.064.

Finally, we evaluated whether the latency between the primary surgery and adjuvant chemotherapy influences the outcome in unexpected EOC. Figure 3A,B suggested that women who had latency >28 days had similar DFS (*p* = 0.31, log‐rank test) compared to those without adjuvant chemotherapy, those who underwent staging surgery alone, or those who had chemotherapeutic latency ≤28 days. However, a trend of poor OS (*p* = 0.064, log‐rank test) was noted for those receiving adjuvant chemotherapy >28 days compared to those without adjuvant chemotherapy, who underwent staging surgery alone, or chemotherapeutic latency ≤28 days, though it did not reach significance. Thus, our results indicate that chemotherapeutic latency was not a confounding factor for outcome in unexpected EOC.

## 4 DISCUSSION

Our results showed that tumor size and subsequent staging surgery were different between the women who underwent LSC versus LAPA. Primary surgical method was not a confounding factor for the DFS or OS of unexpected early‐stage ovarian cancer. In unexpected EOC, the only prognostic factor for poor DFS was advanced FIGO stage while there were multiple independent prognostic factors for poor OS, including advanced FIGO stage, mucinous histology, and high‐grade (Gr III) tumor. Surgical latency between primary surgery and staging surgery >28 days was also a poor prognostic factor. However, the chemotherapy latency was not a risk factor.

The primary operative method influencing the outcome of unexpected ovarian cancer is still controversial. In apparent early‐stage ovarian cancer, the NCCN and ESMO‐ESGO consensus recommendations suggest laparotomy to be the current standard for staging surgery.^17^ Studies have shown that primary LSC adnexectomy is a feasible initial operation for patients with adnexal masses, even early‐stage ovarian malignancies, without increasing the rate of recurrence.^8^, ^18^ Bogani et al. suggested that minimally invasive surgery (MIS) and open surgery have similar survival outcomes in patients with ovarian cancer.^19^ Melamed et al. also reported that surgical staging with LSC is not inferior to open surgery in terms of the survival of stage I ovarian cancer.^20^ Our study demonstrated that LSC as the primary surgical method does not result in poorer outcomes from unexpected ovarian cancer. However, Matsuo et al. presented that MIS is associated with an increased risk of capsular rupture and is further associated with inferior survival compared to no tumor rupture in both MIS and open surgery.^21^ Currently, there is no randomized controlled trial evaluating the oncological outcomes in early‐stage ovarian cancer. Therefore, a randomized clinical study is necessary to evaluate whether LSC influences the outcome of patients with early‐stage ovarian carcinomas.

The first concern of LSC as the primary surgical procedure for ovarian cancer is the iatrogenic upstaging of ovarian cancer because of tumor rupture during surgery. Yuen et al. reported similar tumor rupture rates of benign ovarian masses between LSC and LAPA in a randomized trial.^22^ Shiota et al. suggested that the rupture rate of benign ovarian tumors significantly increased with LSC compared to LAPA when tumors were larger than es 10 cm.^23^ The gas system (CO_2_) during LSC is another potential hazard of cancer cell spread during surgery if tumor rupture occurs.^24^ In our series, LSC resulted in a higher incidence of further comprehensive staging surgery than LAPA (56.9% vs. 25.5%, *p* = 0.001). This implied that surgeons and/or patients were less confident based on the results of LSC surgery. Upstaging occurs in 31–50% of staging or restaging surgery for patients with unexpected ovarian cancers.^25^ Our series showed similar upstaging rates between LSC and LAPA after comprehensive staging surgery (27% vs. 33%). And according to our results, there were 71.1 (37/52) % and 47.3 (18/38) % of stage I cases with stage IC disease in LSC and LAPA groups in our series. Arlene et al. recommended that all unexpected EOC patients should undergo comprehensive surgical staging due to the identification of occult metastases in a significant number of patients after comprehensive staging surgery.^26^ There is a decreased risk of recurrence in surgical (ovarian cancer with complete surgery that has documentation of staging) stage I cancers compared to clinical (one with incomplete surgery or one that is not diagnosed through surgery) stage I disease without comprehensive surgical staging^26^, ^27^ Thus, we recommend complete staging surgery for all patients with unexpected ovarian cancer regardless of the primary surgical procedure to avoid unexpected advanced‐stage disease. The cancer stage was a poor prognostic factor for the outcome of unexpected ovarian cancer.^28^, ^29^ Tumor rupture during surgery could upstage the ovarian cancer from stage IA to IC, moreover to stage III. Vergote et al. reported that the risk of spontaneous tumor rupture or rupture during surgery was 2.65 or 1.65 for DFS.^30^ LSC was shown to have a higher possibility of tumor rupture during surgery, which led to disease upstaging and then a higher incidence of disease relapse and mortality.^29^, ^30^ Avoiding tumor rupture during surgery is important; therefore, we recommend using containers, such as an endoscopic bag to completely pack the tumor, and then suction out the tumor content within the bag without leakage, which may result in intraperitoneal spread during LSC.

Histological type was another poor prognostic factor for the outcome of unexpected ovarian cancer. Mucinous histology had a lower response to platinum‐based chemotherapy than serous histology.^31^ Cho et al. reported that mucinous‐type early‐stage ovarian carcinoma did not influence patient outcome if restaging surgery was performed.^32^ However, the mucinous histology showed poorer outcome compared to serous and clear cell histologies (Table 3). Mucinous histology always had a specific characteristic of large multicystic septa on preoperative sonography. Therefore, we recommend oophorectomy without tumor rupture during surgery for multi‐cystic tumors favoring mucinous histology.

Tumor grade was also a prognostic factor for the outcome of unexpected ovarian cancer. Tu et al. addressed that unfavorable tumor grade resulted in a higher incidence of occult residual disease.^33^ They designed a scoring system, quality of initial surgery (QOIS), to determine whether restaging surgery should be performed at each initial surgical procedure according to descriptions in the surgical record.^33^ NCCN guideline defines incomplete surgery with the following standards: (1) intact uterus; (2) intact adnexa; (3) retained omentum; (4) incomplete documentation of staging; or (5) residual disease. The QOIS score had a full score of 14 points with >7 as high and ≤7 as low. They found that patients with high QOIS needed more restaging surgery than those with low QOIS. In addition, comprehensive staging surgery resulted in better DFS and OS compared to chemotherapy in patients with an unfavorable (high) tumor grade.^33^ However, the outcome of patients with favorable (low) tumor grade was not different when undergoing comprehensive staging surgery or chemotherapy directly.^33^ Our survey also revealed that high tumor grade was an independent prognostic factor due to its higher incidence of occult residual disease and lymph nodal metastasis.^33^, ^34^

Surgical latency between primary operation and comprehensive staging surgery was a prognostic factor for unexpected ovarian cancer. Several studies reported a range of mean delay between primary surgery and staging surgery from 33 to 88 days.^8^, ^15^, ^35^, ^36^, ^37^ In the present study, the mean interval between the primary surgery and staging surgery was 29 days, and an interval delay ≤28 days had significantly better outcome than ≥28 days. Lehner et al. reported that patients had a high possibility of advanced (stages IIB‐IV) diseases if the interval between staging laparotomy and primary LSCwas >16 days compared to <16 days or immediate staging laparotomy.^36^ Muzii et al. also proposed making every effort to minimizing the surgical latency in unexpected ovarian cancer.^15^ We recommend staging surgery for unexpected ovarian cancer after primary surgery, especially LSC surgery.

Adjuvant chemotherapy was always used for ovarian cancer after staging or cytoreductive surgery. Adjuvant chemotherapy has been demonstrated to improve the outcome and reduce the recurrence of high‐risk early‐stage and advanced‐stage ovarian cancer.^38^ Seagle et al. reported that chemotherapy longer than 35 days after surgery is associated with a 7% increased hazard of death compared to <35 days.^39^ Our study also demonstrated that chemotherapy >28 days after primary LSC or LAPA tended to result in increased recurrence and mortality compared to <28 days. Therefore, we recommend adjuvant chemotherapy after primary surgery alone or comprehensive staging surgery in unexpected ovarian cancer less than 28 days after surgery.

Intraoperative frozen pathology can provide an immediate diagnosis of ovarian tumors and decrease the incidence of unexpected ovarian malignancies. For most ovarian cancers or those that are suspected to be ovarian cancer in our department, intraoperative frozen pathology is sent.^40^ However, we still have some unexpected ovarian cancers. Diagnosing ovarian tumors is often challenging. Traditionally, definitive diagnosis requires histological evaluation of the entire removed adnexa. The alternative methods for diagnosing ovarian malignancy include needle aspiration and image‐guided core biopsy.^41^, ^42^ Preoperative serum markers, including CA‐125 and/or HE4, and imaging studies, including sonography, CT, and MRI could provide the differential diagnosis for ovarian tumors.^43^ It is important to consider the possibility of ovarian LMP and malignancies before surgery. Intraoperative frozen pathology is a highly accurate, sensitive, and specific method to help diagnose unexpected ovarian cancer. It can also help decrease the incidence of a second surgery for ovarian cancer. Because intraoperative frozen pathology is time‐consuming, requires a well‐prepared facility and well‐experienced gynecologic pathologists. So it could not be a standard of care for all ovarian tumors during surgery. We still recommend intraoperative frozen pathology when malignancy is suspected during surgery to avoid a delay in comprehensive staging surgery and to convert LSC to LAPA when frozen pathology reveals ovarian malignancy.

The previous related studies on unexpected ovarian cancers concentrated on the complications of the conversion of a simple surgery to an advance staging surgery during laparoscopy, such as the addition of trocar injuries, injury of vena cava and aorta, as shown in Table 4. They did not have detailed gynecological oncology‐related parameters on the outcome of these patients. A strength of our study was that we recruited patients who received diagnosis and treatment from one medical institution. The limitations of our study are inherent to its retrospective design, including selection bias and not all of the patients underwent complete staging surgery after primary surgery. However, it is difficult and unethical to design a randomized clinical trial to evaluate the influence of different surgical procedures between MIS and laparotomic surgeries on the treatment of unexpected ovarian cancer, the effect of comprehensive staging surgery, and the time lapse time between primary surgery and staging surgery. Recruiting more patients with unexpected ovarian cancer is necessary to answer the important unanswered issues.

**TABLE 4:** Summary of unexpected ovarian cancer studies

	Incidence	No. of total cases	No. of malignant cases	Upstage due to tumor rupture	Mean time to 2nd surgery (day)	Early/advanced cancer patients
Maiman 1991^35^	NA	NA	42	NA	4.8 weeks	NA
Childers 1996^11^	13.8%	138	19	NA	NA	NA
Canis 1997^12^	10.9%	230	NA	NA	NA	NA
Dottino 1999^13^	13.1%	160	21	NA	NA	NA
Biran 2002^14^	18.9%	95	NA	NA	NA	NA
Matsushita 2014^8^	1.5%	884	13	69.2%	88.9 (39–182)	NA
Saito 2014^18^	2.0%	487	10	NA	NA	NA
Cheng 2022	4.7%	2383	112	55/90	59.4 (5–287)	98/14

Abbreviations: NA, not available; No., number.

In conclusion, unexpected ovarian cancer is a difficult issue for surgeons. Surgeons should carefully evaluate patients with an adnexal mass and keep in mind the possibility of ovarian cancer. Regardless of recent developments in the management of diseases due to technological developments, stage is still the most important prognostic factor in ovarian cancer. Staging surgery is crucial for ovarian cancers. Another advantage of staging surgery is that it could determine whether adjuvant therapies are needed to improved patient survival. Comprehensive staging surgery should be considered in all unexpected ovarian cancer patients and be performed as soon as possible after the exact diagnosis of ovarian cancer.

## AUTHOR CONTRIBUTIONS

**Ching‐Yu CHENG:** Conceptualization (equal); data curation (equal); formal analysis (equal); methodology (equal); writing – original draft (equal). **Heng‐Cheng Hsu:** Data curation (equal); formal analysis (equal); methodology (equal); writing – review and editing (equal). **Yi‐Jou Tai:** Data curation (equal); formal analysis (equal); methodology (equal); writing – review and editing (equal). **Ying‐Cheng Chiang:** Formal analysis (equal); methodology (equal); writing – review and editing (equal). **Yu‐Li Chen:** Formal analysis (equal); methodology (equal); writing – review and editing (equal). **Wen‐Fang Cheng:** Conceptualization (equal); formal analysis (equal); investigation (equal); methodology (equal); project administration (equal); supervision (equal); writing – original draft (equal).

## FUNDING INFORMATION

This work was not supported by any grant.

## CONFLICT OF INTEREST

No potential conflicts of interest were disclosed.

## ETHICS APPROVAL

This study was approved by the institutional Research Ethics Committee at the National Taiwan University Hospital (approval No.201904094RINC). All of the patients' data were fully anonymized before we accessed them and the Research Ethics Committee waived the requirement for informed consent.

## Data Availability

The data are not publicly available due to privacy or ethical restrictions but can be provided upon request from the corresponding author.
